# Supplementary material for: Gene Variant Related Neurological and Molecular Biomarkers Predict Psychosis Progression, with Potential for Monitoring and Prevention
Source: Int J Mol Sci. 2024 Dec 12;25(24):13348. doi: 10.3390/ijms252413348 (PMC11677369; doi:10.3390/ijms252413348)
Supplement: Supplementary file 1 [file ijms-25-13348-s001.zip › ijms-3262037-supplementary.pdf]

Supplementary Material

Gene Variant Related Neurological and Molecular Biomarkers Predict Psychosis Progression, with Potential for Monitoring and Prevention.

Correspondence: Prof/Dr Stephanie Fryar-Williams MBBS BSc. FRANZCP-
University of Adelaide, Director Youth in Mind Research Institute, Box 640, Unley Annexe,
Mary St, South Australia, 5061. Phone +61 411310449.

E mail: stephanie.fryar-williams@adelaide.edu.au

Legend

MTHFR C677T gene	gene, coding for 5,10-methylenetetrahydrofolate reductase enzyme
MTHFR enzyme	5,10—methylenetetrahydrofolate reductase enzyme.
5-MTHF	5—methylatetrahydrofolic acid (active form of folate)
5-HIAA	5-hydroxyindoleacetic acid
AD	adrenaline
DA	dopamine
NA	noradrenaline
HVA	homovanillic acid
VMA	vanillylmandelic acid
MHPG	4-hydroxy-3-methoxyphenylglycol
GSH	glutathione (reduced, active antioxidant form)
FAD	flavin mononucleotide
FAD	flavin adenine dinucleotide
Cu	Cu ++ — Free (unbound) copper
Zn	zinc
B6 (PLP)	vitamin B6 (activated form pyridoxal-5-phosphate)
Vitamin D	activated 25-OH form of vitamin D
BPRS	Brief Psychiatric Rating Scale
Case-ness	DSM—diagnostic identification for schizophrenia and schizoaffective disorder
CBS	cystathionine beta synthase.
COMT	catechol-O-methyltransferase
DAO	Diamine oxide
DBH	dopamine beta-hydroxylase
DMG/ TMG	Dimethylglycine / trimethylglycine
DNMT1	DNA methyltransferase 1
B2	vitamin B2 (riboflavin)
FAD	flavin dinucleotide
FMN	flavin mononucleotide
GSH	reduced (active) glutathione

GSSH	oxidised form of glutathione
HCY	homocysteine
HO	haeme oxidase
HPL	hydroxyhaemopyrroline-2-one
HPLC	high-pressure liquid chromatography
KP	kynurenine acid pathway; (KYNA—Kynurenic acid)
MAO	monoamine oxidase
MHMA	Methyl hydroxy mandelic acid
MS	methionine Synthase
OR	Odds Ratio
PANSS	Positive and Negative Syndrome Scale
Peak 1	first HPLC elution peak in urine riboflavin analysis = unidentified riboflavin co-analyte (presumed metabolite)
Peak 2	second peak in riboflavin HPLC urine analysis = riboflavin (standard)
PPV	positive predictive value; NPV—negative predictive value
SAH	S-adenosyl-homocysteine
SAHH	S-adenosyl homocysteine hydrolase enzyme
SAMe	S-adenosylmethionine
SHMT	Serine hydroxymethyl transferase
SOD	Superoxide dismutase.
TP	Tryptophan pyrrolase
TSP	Trans-sulfuration pathway
ASOP	Threshold speed of auditory processing
VSOP	Threshold speed of visual processing
CW age diff %	Competing words as a percentage of age difference (representative of dichotic listening performance)

Contents

S 1. Inclusion criteria. 4
S 2. Exclusion criteria 4
S 3. Rating measures 5
S 4. Assays by licensed Laboratories*, including details of urine riboflavin analysis…....6
S 5. Visual Assessments* 11
S 6. Auditory Assessments* 12
S 7. Case, control recruitment 13
S 8. Data characteristics 13
a. Patient characteristics for age (years) in relationship to duration of illness. 13
b. Data characteristics related to risk factors for functional psychosis. 14
c. Medication profile for cases (STATA analysis). 14
d. Missing data 15
S 9. Access details for expanded background data collection and analysis methods…....15
S 10. *MTHFR* C677T polymorphism is unrelated to case-ness of schizophrenia or schizoaffective disorder in the full data set. 16
S11. Mean duration of Illness (DOI) sorted by *MTHFR* C677T variant. 16
S12. ROC and Odds Ratio results for single and compound variables related to the *MTHFR* 677 CC variant....17
S13. ROC and Odds Ratio results for single and compound variables related to the *MTHFR* 677 TT variant....21
S 14. ROC and Odds Ratio results for single and compound variables related to the *MTHFR* 677T CT variant....25
S 15. Linear regression analyses for DOI, within three *MTHFR* C677T variants 28
S 16. Spearman's correlates for DOI within different *MTHFR* C677T variants. 34
S 17. Ranked significant DOI correlates across the three separate *MTHFR* C677T variants. 44

S 1. Inclusion criteria.

Inclusion Criteria	Method
Orientated in time, place and person. Non-detained under Mental Health Act Capacity to consent	Clinical examination and case-note examination
Diagnosis of schizophrenia or schizoaffective disorder, in accord with DSM-IV-R classification criteria, checked on recruitment enrolment against DSM-IV-R symptom checklist.	American Psychiatric Association: Diagnostic and Statistical Manual of Mental Disorders. (4th Ed. Rev) (DSM IV-R). American Psychiatric Association 1994.
Absence of tardive dyskinesia, instability in ocular-muscle function, rigidity or tremor in neck, shoulder, forearm or hand.	Simpson GM, Angus JWS. A rating scale for extrapyramidal side effects. Acta Psychiatry Scand. 1970; 212 (44):11–9.
Zuclopenthixol, Modecate, Amisulpride Risperidone, Quetiapine, sodium valproate.	Treatment chart and case-note examination
Risk factors of interest: such as family history of mental illness, history of emotional or physical abuse, developmental disorder, learning disorder or subclinical head injury.	Case-note examination and personal inquiry.

S 2. Exclusion criteria

Exclusion Criteria	Method
Lack of orientation in place, person or time. Detained under Mental Health Act Lacking consent capacity	Clinical examination and case-note examination
Alternative diagnosis	American Psychiatric Association: Diagnostic and Statistical Manual of Mental Disorders. (4th Ed. Rev) (DSM IV-R). American Psychiatric Association 1994.
Unstable pharmacotherapy over assessment period.	Treatment chart and case-note examination
Extra-pyramidal side effects in ocular, hand, forearm and shoulder	Clinical examination; Simpson GM, Angus JWS. (1970) A rating scale for extrapyramidal side effects. *Acta Psychiatrica Scandinavica*, **212** (44), 11-19.
Clozapine, Olanzapine, anti-histamines or vitamin therapy	Treatment chart, case-note examination and personal questioning.
Recent or unresolved history or positive test for alcohol or other substance abuse.	Treatment chart, case-note examination and personal questioning

Upper respiratory tract infections	Treatment chart, case-note examination and personal questioning
Intellectual disability or clinically documented or descriptive history of head injury with unconsciousness or hospitalisation.	Treatment chart, case-note examination and personal questioning.
Visual fixation disparity (phoria or tropia)	Alternate cover test. Riordan-Eva P. Cunningham Jr. ET. Vaughan & Asbury's General Ophthalmology. 18th ed. Lange Medical Book. McGraw-Hill; 2011. New York:
Visual and hearing acuity disability	Case-note examination and personal questioning Sussex Vision test of near vision. Near vision test card SNT-3000-L, 2009–2011.Snellen H, 1860, 'Snellen Chart', Sussex Vision International Ltd.<br>Maico Diagnostics: Operating Instructions MA 40, Diagnostic GmbH, 2005 Salzufer 13/14 D-10583, Berlin, Germany. Available from: www.audiometrics.net/resources/MA40E.pdf

S 3. Rating measures

**Functional Rating Scale**	**Citation**
The Brief Psychiatric Rating Scale (BPRS)	Overall JE and Gorham DR (1962) The Brief Psychiatric Rating Scale. *Psychological Report* **10**(3): 799-812.
Positive and Negative Syndrome Scale for schizophrenia (PANSS)	Kay SR, Fiszbein A and Opler LA (1987) The positive and negative syndrome scale (PANSS) for schizophrenia. *Schizophrenia Bulletin* 13(2): 261-276.
Clinical Global Impression of Severity (CGI)	Guy W (1976) Clinical global impressions [CGI]. *ECDEU assessment manual for psychopharmacology.* National Institute of Mental Health, Early Clinical Drug Evaluation, Psychopharmacology Research Branch, Rev. Rockville, MD, U.S.
Global assessment of Function (GAF)	Frances A, Incus HA and First MB (1994) *Diagnostic and Statistical Manual of Mental Disorders*, 4th edition, American Psychiatric Association, Washington, DC.
Social and Occupational Functioning Assessment Scale (SOFAS)	Goldman HH, Skodol AE and Lave TR (1992) Revising axis V for DSM-IV: a review of measures of social functioning. *American Journal Psychiatry* **149**(9): 1148-1156.

S 4. Assays by licensed Laboratories*, including details of urine riboflavin analysis.

**Gene variant detection**	**All fasting blood samples, collected between 9 and 11 am daily. By arrangement, transported directly to the laboratory with no storage.**	**Licensed Laboratory and /or specific analysis method Citation Reference**<br>(* National Association of Testing Authorities (NATA) https://nata.com.au/)
MTHFR Ala222Val (C677T) methyl tetrahydrofolate reductase polymorphism (homo-zygous (TT) point mutation)<br>wild type (CC) sequence and<br>heterozygote (CT) DNA with wild type and<br>mutant strands.	Roche Diagnostics Light-Cycler 480 kit. Using TecnoBiol reagents, Sigma probes and primers on Roche LC480 analyser. Using TecnoBiol reagents, Sigma probes and primers on Roche LC480 analyser. https://lifescience.roche.com/global/en/products/others/lightcycler-480-control-kit-358360.html<br>(access date 25/11 24)<br>LightCycler® FastStart DNA Master HybProbe is an easy-to-use hot start reaction mix for sensitive PCR applications using HybProbe probes as detection format. It detects SNP and mutation detection (and can also be used in two-step RT-PCR). The HybProbe probes consist of two different short oligonucleotides that bind to an internal sequence of the amplified fragment, during the annealing phase of the amplification cycle. The basic steps of DNA detection by HybProbe probes during real-time PCR on the LightCycler® System are available at https://lifescience.roche.com/global/en/products/others/lightcycler-faststart-dna-master-hybprobe-355132.html.<br>(access date 25/11/24)	Douglass Hanly Moir Pathology. Locked Bag 145, North Ryde NSW 1670. https://www.dhm.com.au/<br>(access date 25/11/24)
**Neuro-Biochemistry**	**All fasting blood samples, collected between 9 and 11 am daily. By arrangement, transported directly to the laboratory with no storage.**	**Licensed Laboratory and /or specific analysis method Citation Reference**<br>(* National Association of Testing Authorities (NATA) https://nata.com.au/)
Vitamin D (25-OH)	Diasorin Liason assay kit, for use on the Liaison platform. (nmol/L)<br>Diasorin Australia Pty Ltd. Macquarie Park, NSW 2113, Australia.	Clinpath Laboratories, 19 Fullarton Rd, Kent Town. South Australia 5067<br>+61 8 8366 2000.

Serum total Vitamin B12	Competitive Electro-chemiluminescent Immunoassay. Roche Modular E 170 Automated Immunoassay Analyser, using Roche Vitamin B12 Reagent. (nmol/L). Roche Diagnostics Australia Pty. Limited North Ryde, NSW 2113, Australia	Clinpath Laboratories, 19 Fullarton Rd, Kent Town. South Australia 5067 +61 8 8366 2000.
Plasma Red Cell Folate	Competitive Electro-chemiluminescent Protein Binding Assay, using Roche Modular E 170, using Roche Folate Red Blood Cell (RBC) Reagent and Roche Folate RBC Haemolysing Reagent on Automated Immunoassay Analyser. (nmol/L). Roche Diagnostics Australia Pty. Limited, North Ryde, NSW 2113, Australia.	Clinpath Laboratories, 19 Fullarton Rd, Kent Town. South Australia 5067 +61 8 8366 2000.
Serum Vitamin B6 (Pyridoxal- 5′-phosphate coenzyme form)	Whole blood High Pressure Liquid Chromatography with fluorescent detection. Chromsystems Vitamin B6 in Whole Blood High pressure Liquid Chromatography Reagent Kit. Waters Alliance 2695 Separations Module. Waters 474 Fluorescence Detector(nmol/L). Chromsystems Instruments & Chemicals 82166 Gräfelfing, Munich, Germany.	Sullivan Nicolaides Pathology 143 Whitmore St, Taringa. Queensland 4068, Australia. +61 7 337 8666
Serum Copper	Flame Atomic Absorption Spectrophotometry. Varian AA-240FS (umol/L). https://www.agilent.com/en/product/atomic-spectroscopy/atomic-absorption/flame-atomic-absorption-instruments/240fs-aa (last accessed 25/11/24).	Douglass Hanly Moir Pathology 14 Griffnock Avenue, Macquarie Park. New South Wales 2113. Australia. +61 2 98555222.
Plasma Red Cell Zinc	Inductively coupled plasma mass spectroscopy (ICP-MS), using 6% n-Butanol reagent and Agilent ICP-MS 7500ce analyser (umol/L). https://www.agilent.com/en/product/atomic-spectroscopy/inductively-coupled-plasma-mass-spectrometry-icp-ms (last accessed 25/11/24).	Sullivan Nicolaides Pathology 143 Whitmore St, Taringa. Queensland 4068. Australia. +61 7 337 8666.

Serum Ceruloplasmin	Immunoturbidimetric method, using 6K91-30 Multignet Caeruloplamin Kit and Abbott Architect ci16000 analyser. (g/L). https://www.corelaboratory.abbott/int/en/offerings/brands/architect/architect-c16000.html (last accessed 25/11/24).	Douglass Hanly Moir Pathology. Griffnock Avenue, Macquarie Park. New South Wales 2113. +61 2 98555222.
Percentage Free Copper/Red Cell Zinc	Percentage of free copper in the serum calculated by an equation based on the molecular and atomic weights of ceruloplasmin and copper (one ceruloplasmin molecule binds to six copper atoms). The ratio of the percentage free copper to red cell zinc was calculated as "percentage free copper" / "Red cell zinc umol/L".	Calculated by authors.<br>Method in: Walsh, W.J.; Isaacson, H.R.; Rehman, F.; Hall, A. Elevated blood copper/zinc ratios in assaultive young males. *Physiology & behavior* **1997,** 62, 327-9.
Plasma homocysteine	Ice transported EDTA sample. Competitive Chemiluminescent Immunoassay, using Seimens Homocysteine reagent on Seimens Advia centaur Automated Immunoassay (umol/L). https://www.internationaldiagnostic.com/product/siemens-advia-centaur-xpt-immunoassay-system/?srsltid=AfmBOorZDX9UR6xqpDyOfMpGJ6pDHa0ts7lwt08_lzZnPnVpN285evEk (last accessed 25/11/24).	SA Pathology. Adelaide, South Australia 5000. Australia. +61 8 8222 3000.
Serum histamine	Beckman Coulter Radio Immunoassay, using Beckman Coulter R.I.A. Kit on Perkin Elmer Wizard 1470 Automated Gamma Counter. (umol/L). PerkinElmer Norge AS - 965 612 246 - Oslo Norway. https://content.perkinelmer.com/ (last accessed 25/11/24).	Sullivan and Nicolaides.143 Whitmore St, Taringa. Queensland 4068. Australia. +61 7 337 8666.

**Urine Tests Neurotransmitters**	**Method, Analyzer, Reagents.**	**Laboratory/Reference**
Biogenic amines: Dopamine, Noradrenaline and Adrenaline,	Spot-baseline (fasting) urinary neurotransmitter testing (second void morning), snap-frozen to minus 30 degrees and analysed by mass spectrometry, using nanomols per millimol of urinary creatinine as a standard.	SA Pathology, Adelaide, South Australia. Australia.<br>Method: Whiting MJ. 2009. Simultaneous measurement of urine metanephrines and catecholamines by liquid chromatography with tandem mass spectrometric detection. *Annals of Clinical Biochemistry*, **46**:129-136

Creatinine	Spot urine specimen from the same void as biogenic amines, expressed in (millimols per Litre)	SA Pathology, Adelaide SA. 5000. Australia.<br><br>Method as above.
Oxidative stress: Urinary hydroxyhemopyrroline-2- one	Fasting urine sample collected whilst patient at rest, separated from blood drawing by minimum of 2 hours.	Applied Analytical laboratories, Meadowbrook, Queensland. Australia. https://www.apanlabs.com/<br><br>(last accessed 25/11/24).
Urine Riboflavin ug/L	Ultra performance liquid chromatography (HPLC), with fluorometric detection of the eluted riboflavin Peak (1) riboflavin metabolites and peak (2) = riboflavin.	Lipid analysis laboratory Waite Agricultural Research Institute. Adelaide SA 5000, Australia. (Supplementary Materials, Section S4) Fryar-Williams, S.; Tucker, G.; Strobel, J.; Huang, Y.; Clements, P. Molecular Mechanism Biomarkers Predict Diagnosis in Schizophrenia and Schizoaffective Psychosis, with Implications for Treatment. *Int. J. Mol. Sci.* **2023**, *24*, 15845. https://doi.org/10.3390/ijms242115845<br><br>(last accessed 25/11/24).

HPLC elution phenotype for riboflavin where height Peak 2 > Peak 1.

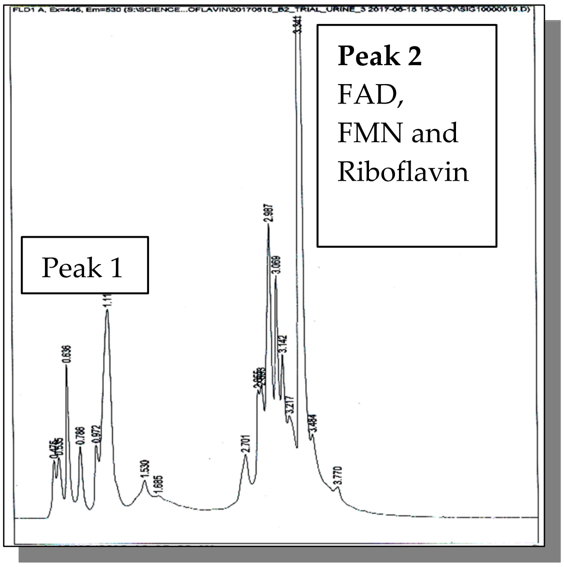

HPLC elution phenotype for riboflavin where height Peak1 >Peak 2.

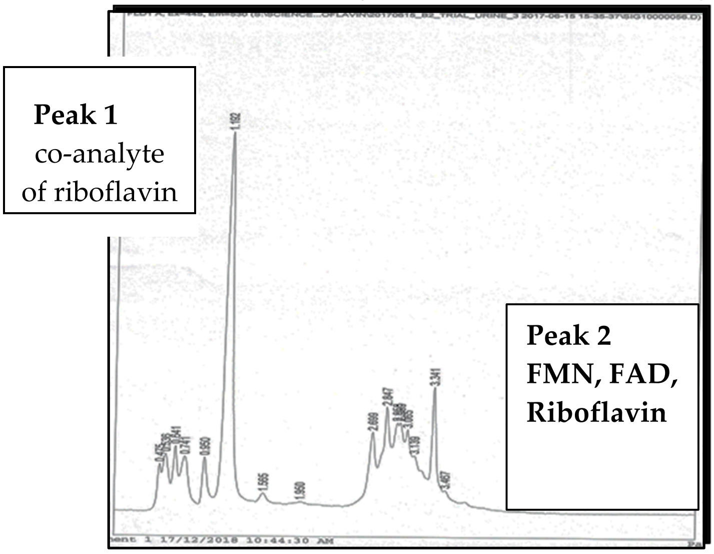

S 5. Visual Assessments *

Assay/Assessment<br>Visual	Method	*Reference for licensed test
Near vision acuity test	Sussex Vision test of near vision. Near vision test card SNT-3000-L, 2009–2011.	Sussex Vision International Ltd. (35) http://sussexvision.co.uk/index.php/near-tests/reading-tests.html<br>(last accessed 25/11/24)
Visual (symbol) span	Sussex Vision test of near vision. Near vision test card SNT-3000-L, 2009–2011.	Sussex Vision International Ltd. (35) http://sussexvision.co.uk/index.php/near-tests/reading-tests.html<br>(last accessed 25/11/24)
Distance vision (Binocular distance vision acuity)	Sussex Vision test of near vision. Near vision test card SNT-3000-L, 2009–2011.	Sussex Vision International Ltd. (35) http://sussexvision.co.uk/index.php/near-tests/reading-tests.html<br>(last accessed 25/11/24)
Distance vision (Binocular distance vision acuity)	Right distance vision, then left distance vision, with 20 seconds inter-test interval.	The Snellen-Chart. Alpha Medical Solutions, St Ives, New South Wales 2075, Australia.<br>(H. Snellen, Probebuchstaben zur Bestimmung der Sehschärfe, Utrecht 1862).
Threshold visual speed of processing performance as a percentage of age. Expresses visual processing speed in terms of the visual processing system's relative age). Shortest interval of time a person can notice between the order of presentation of two optical stimuli. Speed of visual order processing increases with age. For adults between the range of 18 and 60 years, the normal range for visual speed of processing is 24 to 72 milliseconds). For adults between the range of 18 and 60 years, the normal range for visual speed of processing is 24 to 72 milliseconds.	Person tested sees two brief flashes of light randomly presented from left-to-right or right-to-left on multiple occasions and must decide which light flash appeared first. The inter-stimulus time interval (ISI) between the flashes is shortened by computer algorithm, if the answer is correct, otherwise it is lengthened. A performance-age rating, is provided, configured against norms-for-age. Performance-age is subtracted from the individual's actual age and the result divided by the age of the test subject is multiplied by 100.	Brain Boy Universal Professional instrument (MediTECH 2010) http://www.meditech.de/fileadmin/download/anleitungen/manual_BUP-neu-03.03.2010.indd-mail.pdf<br>(last accessed 25/11/24)<br>MediTECH® Electronic GmbH<br>Langer Acker 7<br>D-30900 Wedemark, Germany.<br>http://www.meditech.de<br>Telefon: +49-(0)5130) 97778-0<br>Fax: +49-(0)5130) 97778-22<br>Email: service@meditech.de

S 6. Auditory Assessments*

**Assay/Assessment Auditory**	**Method**	***Reference for licensed test**
Reverse digit span (Measures auditory (verbal) working memory) Normal range is 6 to 7	With gaze aversion by listening participant and tester, digits are read in set sequence. The tested participant is asked to repeat them in reverse order. Reported as the absolute number of digits correctly recalled in reverse order.	Subset of Wechsler Adult Intelligence Scale III (Wechsler 1997) Pearson http://www.pearsonclinical.com/psychology/products/100000243/wechsler-adult- intelligence-scale--third-edition-wais-iii.html (last accessed 25/11/24)
Competing words performance for age as a percentage of age Intra-cerebral dichotic listening performance for processing of auditory information Normal ranges vary with age	A voice-over CD and earphones test ability to correctly identify both of two competing-words (CW), delivered separately to the right and left ears. Using this test's normative-for-age database, the difference between each test subject's expected and actual performance-for-age was calculated, and this was then divided by the actual age of the test subject and multiplied by 100.	SCAN-3:A Tests for Auditory Processing Disorders in Adolescents and Adults (Keith 2009) https://www.pearsonclinical.com.au/produc ts/view/315 (last accessed 25/11/24)
Threshold speed of Auditory processing. Speed of auditory processing systems relative to age. Speed of auditory processing reduces with age. For adults in the age range of 18 and 60 years, the normal range for auditory speed of processing is 46 to 72 milliseconds.	Person tested hears two clicks, randomly presented from right to left and left to right side, presented through headphones. By pressing a right or left button, a decision must be made from which side the dual stimulus originates. If the answer is correct, the inter-stimulus interval between flashes (ISI) is shortened, otherwise it is lengthened. The auditory order threshold is the shortest ISI a person can correctly differentiate between two auditory impressions. A read-out of the threshold speed of auditory (order) processing is provided, along with a norm performance-age rating. Auditory speed of (order) processing performance as a percentage of age is calculated by subtracting the norm-for-age from the performance-age, divided by the age of the test subject, multiplied by 100.	Brain Boy Universal Professional instrument (MediTECH 2010) Equivalent, upgraded version available https://www.meditech.de/en_US/shop/2222-gb-set-brain-boy-universal-bbu-english-5190?srsltid=AfmBOoqW2YH_tavEn0SYMD103ZELelBcDJjKNTIzsHDDELdYkgCxyt5G#attr= (Last accessed 25/11/24)

S 7. Case, control recruitment

Cases n =67	Controls n =67
○ Diagnosed schizophrenia and schizoaffective disorder<br>○ drawn from a catchment area population of 22,000.<br>○ Imposition of multiple exclusion criteria restricted eligible cases to 370<br>○ 4 eligible cases of psychosis were allocated a diagnosis of psychosis for investigation<br>○ only twenty five percent of these consented.<br>○ high refusal rate resulted in 89 consenting cases,<br>○ 7 did not reach assessment due to declining mental state and<br>○ 15 were excluded due to the confounding factor of SSRI, SNRI medication.	○ no diagnosis of schizophrenia or DSM diagnosed mental illness, rated for subclinical symptoms<br>○ drawn from an available sample number of 2489.<br>○ randomization and imposition of recruitment exclusion criteria<br>○ low consent rate of 25 per cent,<br>○ total of 72 control participants were recruited.<br>○ 5 of excluded due to failure to meet exclusion criteria on assessment<br>○ matched for age and sex with patients.

S 8. Data characteristics

a. Patient characteristics for age (years) in relationship to duration of illness.

Age group	*n*(duration)	MINIMUM duration	MEAN duration	MAXIMUM duration
15-19	0			
20-24	3	0.5	5.16	10
25-29	8	1	11.25	33
30-34	13	3	10.46	17
35-39	7	7	13	17
40-44	5	12	18.2	24
45-49	8	5	22.37	33
50-54	10	8	24.9	44
55-59	4	7	21.25	46
60-64	3	10	29	47

b. Data characteristics related to risk factors for functional psychosis.

		Schizophrenia		Schizoaffective psychosis		Psychosis FI	
		n	%	*n*	%	*n*	%
FAMILY HISTORY	No	20	66.7	16	55.2	3	75.0
	Yes	10	33.3	13	44.8	1	25.0
DEVELOPMENTAL DISORDER/DELAY	No	9	29.0	14	50.0	2	50.0
	Yes	22	71.0	14	50.0	2	50.0
LEARNING DELAY and DISORDER HISTORY	No	12	36.4	16	53.3	2	50.0
	Yes	21	63.6	14	46.7	2	50.0
HEAD INJURY (subclinical, premorbid)	No	21	70.0	15	51.7	4	100.0
	Yes	9	30.0	14	48.3		
EAR INFECTION	No	18	62.1	18	69.2	4	100.0
	Yes	11	37.9	8	30.8		
OTOSCOPY	No	23	71.9	21	80.8	2	100.0
	Yes	9	28.1	5	19.2		
BONE CONDUCTION ABNORMALITY	No	11	34.4	12	46.2	2	66.7
	Yes	21	65.6	14	53.8	1	33.3

c. Medication profile for cases (STATA analysis).

	n Schizo-phrenia	n Schizo-affective	n Other	Total
ABILIFY	1	1	0	2
AMISULPRIDE	1	1	0	2
CHLORPROMAZINE	1	1	0	2
FLUPENTHIXOL	1	1	0	2
HALOPERIDOL	0	1	0	1
LITHIUM	0	3	0	3
BENZODIAZEPINE	2	2	0	4
PALIPERIDONE	1	0	0	1
QUETIAPINE	2	8	0	10
RISPERIDONE	18	7	3	28

VALPROATE	2	2	0	4
ZIPRAZIDONE	1	1	0	2
ZUCLOPENTHIXOL	4	1	0	5
MODECATE	0	1	0	1
OTHER	1	9	1	11
Total	35	39	4	78
Cases	29	28	4	61

d. Missing data

Domain type	Observed	Missing	Total	% data missing
Laboratory-derived variables	126	2	134	1.5
Visual variables	120	11	134	8.2
Auditory variables	120	10	134	7.5

S9. Access details for expanded background data collection and analysis methods. Fryar-Williams S, Tucker G, Strobel J, Huang Y, Clements P. Molecular Mechanism Biomarkers Predict Diagnosis in Schizophrenia and Schizoaffective Psychosis, with Implications for Treatment. Int J Mol Sci. 2023 Oct 31;24(21):15845. doi: 10.3390/ijms242115845. PMID: 37958826; PMCID: PMC10650772.
Go to: Supporting information in **Supplementary Materials**
Click here for additional data file.(639K, zip)
https://www.mdpi.com/article/10.3390/ijms242115845/s1.
https://www.ncbi.nlm.nih.gov/pmc/articles/PMC10650772/#app1-ijms-24-15845title
National Institutes of Health (NIH) (.gov)
https://www.ncbi.nlm.nih.gov › articles › PMC10650772
(last accessed 25/11/24)

S 10. *MTHFR* C677T polymorphism is unrelated to case-ness of schizophrenia or schizoaffective disorder in the full data set.

mthfrpolymorphism and caseness are unrelated (p=0.924).

```
. tab mthfrpoly case, chi

                    |          case
MTHFR Polymorphism |         0          1 |     Total
-------------------+----------------------+----------
  MTHFR homozygous |         4          3 |         7
MTHFR heterozygous |        31         31 |        62
    MTHFR negative |        32         33 |        65
-------------------+----------------------+----------
             Total |        67         67 |       134

          Pearson chi2(2) =   0.1582   Pr = 0.924
```

S11. Mean duration of Illness (DOI) sorted by *MTHFR* C677T variant.

Duration of illness symptoms in years

```
-> mthfr polymorphism = MTHFR homo TT
   Variable |    Obs    Mean   Std. Dev.   Min   Max
-------------+--------------------------------------------------------
Durationof~s |      7  5.714286   9.357961     0    24
--------------------------------------------------------------------
-> mthfr polymorphism = MTHFR hetero CT
   Variable |    Obs    Mean   Std. Dev.   Min   Max
-------------+--------------------------------------------------------
Durationof~s |     61  9.057377   12.3707     0    47
----------------------------------------------------------------------
-> mthfr polymorphism = MTHFR negative CC
   Variable |    Obs    Mean   Std. Dev.   Min   Max
-------------+--------------------------------------------------------
Durationof~s |     60  7.183333   10.51148     0    44
. mean Durationofillnessyrs
. bysort mthfrpoly:summarize Durationofillnessyrs
---------------------------------------------------
Mean estimation          Number of obs   =     128
-------------------------------------------------------
                    |    Mean   Std. Err.   [95% Conf. Interval]
Durationofillnessyrs |  7.996094  1.003539   6.010272   9.981915
-----------------------------------------------------------------
```

S12. ROC and Odds Ratio results for single and compound variables related to the *MTHFR* 677 CC variant.

Biomarkers considered to be representative of an low methylation phenotype are presented in blue type and biomarkers considered representative of a high methylation phenotype are presented in red. Where no data is given, this is indicative of failure of a given variable to produce a significant biomarker.

**Variable for *MTHFR* 677 CC**	***n***	**Sensitivity %**	**Specificity %**	**PPV %**	**NPV %**	**OR**	**OR *p* value**	**AUC**	**Standard Error**	***p***
**High 5HIAA**	65							0.7557	0.0553	0.0000
**High 5HIAAROC**	65	84.8	59.4	0.9	99.9	8.2	0.001	0.7211	0.0543	0.0000
**DA x 5HIAA**	65							0.7888	0.0561	0.0000
**DA X 5HIAA ROC**	65	84.8	71.9	1.4	99.9	14.3	0.0000	0.7836	0.0513	0.0000
**NA/DA**	65							0.8324	0.0520	0.0000
**NA/DA ROC**	65	69.7	90.6	3.3	99.8	22.2	0.000	0.8016	0.0483	0.0000
**NA/MHMA**	63							0.8187	0.0550	0.0000
**NA/MHMA ROC**	63	84.8	70.0	1.3	99.9	13.1	0.000	0.7742	0.0531	0.0000
**Low B6**	63							0.6930	0.0672	0.0020
**Low B6 ROC**	63	90.6	48.4	0.8	99.9	9.1	0.002	0.6951	0.0526	0.0001
**High Histamine ROC**	65	93.9	28.1	0.6	99.9	6.1	0.030	0.6103	0.0456	0.0078
**Low Vit D**	64							0.6987	0.0652	0.0012
**Low Vit D ROC**	64	75.0	59.4	0.8	99.8	4.4	0.007	0.6719	0.0588	0.0017
**Low red cell**	64							0.6416	0.0699	0.0214

**folate**										
**Low red cell Folate ROC**	64	53.1	78.1	1.1	99.7%	4.0	0.012	0.6563	0.0582	0.0036
**VitB12 / vitamin D High Vit B12/vit D ROC**	64<br>64	87.5	46.9	0.7	99.9	6.2	0.005	0.7031<br>0.6719	0.0644<br>0.0538	0.0008<br>0.0007
**Vit D /vitB12 ROC**	64	87.5	46.9	0.7	99.9	6.2	0.005	0.6719	0.0538	0.0007
**HPL/Creatinine**	65							0.6903	0.0654	0.0018
**High HPL / Creatinine ROC**	65	75.8	56.3	0.8	99.8	4.0	0.010	0.6600	0.0585	0.0031
**HPL/SG**	65							0.6165	0.0706	0.0495
**High HPL/SG ROC**	65	57.6	68.8	0.8	99.7	3.0	0.035	0.6316	0.0603	0.0145
Serum B12	65							0.5218	0.0736	0.3875
Free % Cu /Zn	65							0.5170	0.0742	0.4094
**Free % Cu /Zn ROC**	65	30.3	93.8	2.1	99.7	6.5	0.023	0.6203	0.0461	0.0045
**High Serum B12 ROC**	65	84.8	31.3	0.6	99.8	2.5	0.130	0.5805	0.0523	0.0619
**AD/MHMA**	63							0.8596	0.0468	0.0000
**AD/MHMA ROC**	63	84.8	80.0	1.9%	99.9	22.4	0.000	0.8242	0.0488	0.0000
**AD/NA**	65							0.7083	0.0652	0.0007
**AD/NA ROC**	65	63.6	75.0	1.1%	99.8	5.2	0.002	0.6932	0.0576	0.0004
Plasma homocysteine	64							0.5103	0.0742	0.4448
**High Plasma homocysteine**	64	90.9	25.8	0.6	99.8	3.5	0.088	0.5836	0.0473	0.0386

**ROC**										
[vit B12 X % free Cu] / [Zinc X folate x vit B 6]	62							0.6139	0.0773	0.0703
[vit B12 X % free Cu X Homo cysteine] / [Zinc X folate X vit B6]	62							0.6160	0.0774	0.0670
**vitB12 X Homocysteine X Cu / [Zinc X folate X vit B6 X vitamin D]**	61							0.6462	0.0786	0.0314
**Riboflavin (Peak 1 amplitude - Peak 1 amplitude)**	61							0.6086	0.0728	0.0679
Urine Creatinine	65							0.5578	0.0719	0.2107
Urine B2/creatinine level	61							0.5215	0.0754	0.3886
Urine B2/creatinine ROC	61	71.0	46.7	0.6	99.7	2.1	0.158	0.5882	0.0621	0.0778
**SIR Index**	65							0.9664	0.0303	0.0000
**Low GAF**	58							0.9994	0.0006	0.0000
**Hostility**	65	42.4	100.0	.	99.7	30.8	0.000	0.7121	0.0437	0.0000
**Suicidality**	65	48.5	100.0	.	99.8%	39.1	0.000	0.7424	0.0442	0.0000

**Family history positive**	61	48.3	75.0	0.9	99.7	2.8	0.062	0.6164	0.0612	0.0286
**Developmental delay/disorder**	62	73.3	100.0	.	99.9	106.6	0.000.	0.8667	0.0411	0.0000
**Learning disorder history**	65	60.6	96.9	8.1	99.8	47.7	0.000	0.7874	0.0459	0.0000
Head injury	60	24.1	90.3	1.1	99.6	3.0	0.145	0.5723	0.0486	0.0684
**Ear infection history**	57	32.0	87.5	1.1	99.6	3.3	0.082	0.5975	0.0561	0.0411
**History of Abuse**	65	27.3	100.0	.	99.7	15.7	0.0024	0.6364	0.0394	0.0003
Bone conduction abnormality	60	57.1	56.3	0.6	99.7	1.7	0.302	0.5670	0.0652	0.1521
Otoscopy abnormality	59	14.8	75.0	0.3	99.5	0.5	0.338	0.5509	0.0522	0.1648

**Polymorphism-free MTHFR 677CC**	*n*	**AUC**	*p*	**OR**	*p*	**Lower**	**Upper**
(Using Logistic regression for Odds Ratio (risk estimate) and confidence intervals (CI's)).						95% CI	95% CI
VSOP AgeAdd% ROC (visual speed of processing delay)	57	0.855	0.000	72	0.000	8.349	620.882
Low Visual span ROC	58	0.8293	0.000	23.80	0.000	5.956	95.373
Abnormally high score on Distance Vision R ROC	60	0.6830	0.001	4.644	0.006	1.562	13.812
Low reverse, digit span score ROC	59	0.7679	0.000	16.433	0.000	3.963	68.146

Competing Words % difference ROC (for dichotic listening disorder)	60	0.7746	0.000	15.909	0.000	3.914	64.659
ASOP agediff% ROC (auditory speed of processing delay)	57	0.73314	0.000	12.303	0.001	2.953	51.261

S 13. ROC and Odds Ratio results for single and compound variables related to the *MTHFR* 677 TT variant.

Biomarkers considered to be representative of a low methylation phenotype are presented in blue type and biomarkers considered representative of an high methylation phenotype are presented in red.
Where no data is given is indicative of failure of a given variable to produce a significant biomarker.

Variable	*n*		Speci ficity %	PPV %	NPV %	OR	OR p value	AUC	Standard Error	*p*
DA ROC	7					13.4		1.0000		0.0000
5HIAA	7							0.5000	0.2635	0.5000
5HIAAROC	7	33.3	100.0	100.0	99.7	.	.	0.6667	0.1667	0.1587
NA/DA	7							0.4167	0.2500	0.3695
NA/DA ROC	7			0.6	99.6	1.5	0.810	0.5417	0.2083	0.4207
AD/NA	7							0.667	0.1667	0.0228
AD/NA ROC	7	0.0	75.0	0.0	99.4		.	0.875	0.1250	0.003
NA/MHMA	7							0.5833	0.2500	0.3695
NA/MHMA ROC	7	33.3	100.0	100.0	99.7	.	.	0.6667	0.1667	0.1587
Free Cu Zn	7							0.9167	0.0833	0.0000
Free Cu Zn ROC	7	66.7	75.0	1.2	99.8	6.0	0.287	0.7083	0.2083	0.1587

AD/MHMA	7							0.4167	0.2500	0.3695
AD/MHMA ROC	7	66.7	50.0	0.6	99.7	2.0	0.661	0.5833	0.2205	0.3528
vitB12/vitD	7							0.3333	0.2357	0.2397
vitB12/vitD ROC	7	33.3	100.0	100.0	99.7	.	.	0.6667	0.1667	0.1587
Plasma HCY	7							0.7083	0.2394	0.1921
Pl. HCYROC	7	66.7	75.0	1.2	99.8	6.0	0.287	0.7083	0.2083	0.1587
Histamine	7							0.5417	0.2917	0.4432
Low histamine (<=0.35)	7	33.3	100.0	100.0	99.7	.	.	0.6667	0.1667	0.1587
High Histamine (>=0.59)	7	66.7	25.0	0.4	99.4	0.7	0.810	0.4583	0.2083	0.4207
Histamine ROC	7	66.7	75.0	1.2	99.8	6.0	0.287	0.7083	0.2083	0.1587
Vit D	7							0.3333	0.3333	0.3085
Vit D ROC	7	33.3	100.0	100.0	99.7	.	.	0.6667	0.1667	0.1587
Red cell folate	7							0.5833	0.2500	0.3695
Folate ROC	7	66.7	75.0	1.2	99.8	6.0	0.287	0.7083	0.2083	0.1587
Serum B12	7							0.5833	0.2500	0.3695
B12 ROC	7	100.0	50.0	0.9	100.0	.	.	0.7500	0.1443	0.0416
B6	7							0.3333	0.2357	0.2397
B6 ROC	7	33.3	100.0	100.0	99.7	.	.	0.6667	0.1667	0.1587
HPL/Creatinine	7							0.9167	0.0833	0.0000
HPL/ Creatinine ROC	7	100.0	75.0	1.8	100.0	.	.	0.8750	0.1250	0.0013
HPL/SG	7							0.8333	0.1667	0.0228
HPL/SG ROC	7	100.0	75.0	1.8	100.0	.	.	0.8750	0.1250	0.0013

SIR Index	7							1.0000	0.0000	.
GAF	7							0.0000	0.0000	.
Hostility	7							1.0000	0.0000	.
suicidality	7							0.6667	0.1667	0.1587
Family history	7	0.0	75.0	0.0	99.4	.	.	0.3750	0.1250	0.1587
Developmental delay disorder	7	66.7	75.0	1.2	99.8	6.0	0.287	0.7083	0.2083	0.1587
Learning disorder history	7	66.7	75.0	1.2	99.8	6.0	0.287	0.7083	0.2083	0.1587
Head injury	7	33.3	100.0	100.0	99.7	.	.	0.6667	0.1667	0.1587
Ear infection history	7	33.3	100.0	100.0	99.7	.	.	0.6667	0.1667	0.1587
Bone conduction abnormality	7	66.7	75.0	1.2	99.8	6.0	0.287	0.7083	0.2083	0.1587
Abnormal Otoscopy*	7	100.0	100.0	100.0	100.0	.	.	1.0000	0.0000	.
	*Otoscopy identifies cases perfectly and no other variable has a statistically significant relationship with case-ness.									

**Homozygous MTHFR 677 TT variant**	*n*	**AUC**	*p*	**OR**	*p*	**Lower**	**Upper**
(Using Linear regression for Odds Ratio (risk estimate) and confidence intervals (CI's)).						95% CI	95% CI
VSOP AgeAdd% ROC (visual speed of processing delay)	5	0.75	0.480	0.667	1.000	0.017	-
Low Visual span ROC	7	0.833	0.046	4.449	0.286	0.283	-

Abnormally high score on Distance Vision R ROC	7	0.875	0.003	5.098	0.229	0.37	-
Low reverse, digit span score ROC	7	0.75	0.083	2.225	0.571	0.142	-
Competing Words % difference ROC (for dischotic listening disorder)	6	0.875	0.003	3.303	0.400	0.203	-
ASOPagediff% ROC (auditory speed of processing delay)	5	1.000	0.157	4.000	0.400	0.103	-

S14. ROC and Odds Ratio results for single and compound variables related to the MTHFR 677 CT variant.

Biomarkers considered to be representative of a low methylation phenotype are presented in blue type and biomarkers considered representative of an high methylation phenotype are presented in red. Where no data is given is indicative of failure of a given variable to produce a significant biomarker.

**Variable for *MTHFR* 677CT**	*n*	Sensitivity %	Specificity %	PPV %	NPV %	OR	OR *p* value	AUC	Standard Error	*p*
5HIAA	61							0.6086	0.0683	0.0559
High 5HIAA ROC	61	30.0	93.5	2.1	99.7	6.2	0.028	0.6177	0.0481	0.0072
NA/DA	61							0.7876	0.0580	0.0000
High NA/DA ROC	61	80.0	67.7	1.1	99.9	8.4	0.000	0.7387	0.0566	0.0000
AD/NA	61							0.6032	0.0741	0.0819
AD/NA ROC	61	43.3	87.1	1.5	99.7	5.2	0.012	0.6522	0.0553	0.0030
NA/MHMA	60							0.8144	0.0567	0.0000
High NA/MHMA ROC	60	76.7	80.0	1.7	99.9	13.1	0.000	0.7833	0.0541	0.0000
Free Cu Zn	62							0.6077	0.0726	0.0690
High Free Cu Zn ROC	62	51.6	74.2	0.9	99.7	3.1	0.040	0.6290	0.0606	0.0166
AD/MHMA	60							0.7439	0.0650	0.0001
High AD/MHMA ROC	60	50.0	96.7	6.4	99.8	29.0	0.002	0.7333	0.0493	0.0000
vitB12/vit D	61							0.6118	0.0724	0.0613

High vitB12/vit D ROC	61	76.7	48.4	0.7	99.8	3.1	0.045	0.6253	0.0602	0.0187
Plasma HCY	61							0.5952	0.0736	0.0979
High pl. HCYROC	61	63.3	58.1	0.7	99.7	2.4	0.097	0.6070	0.0635	0.0460
Histamine	62							0.5937	0.0724	0.0978
Low histamine (<=0.35)	62	6.5	90.3	0.3	99.5	0.6	0.643	0.4839	0.0351	0.3232
High Histamine (>=0.59)	62	67.7	35.5	0.5	99.6	1.2	0.788	0.5161	0.0611	0.3961
High Histamine ROC	62	45.2	77.4	0.9	99.7	2.8	0.064	0.6129	0.0593	0.0285
Vit D	61							0.6269	0.0723	0.0396
Low Vit D ROC	61	70.0	58.1	0.7	99.8	3.2	0.030	0.6403	0.0620	0.0118
Red cell folate	62							0.6774	0.0686	0.0049
Low Folate ROC	62	67.7	64.5	0.9	99.8	3.8	0.013	0.6613	0.0611	0.0041
Serum B12	62							0.6098	0.0727	0.0655
High B12 ROC	62	54.8	67.7	0.8	99.7	2.6	0.076	0.6129	0.0623	0.0350
Vitamin B6	59							0.5833	0.0768	0.1390
Low B6 ROC	59	76.7	44.8	0.6	99.8	2.7	0.085	0.6075	0.0612	0.0395
HPL/ Creatinine	61							0.6935	0.0709	0.0032

High HPL/Creatinine ROC	61	66.7	74.2	1.2	99.8%	5.7	0.002	0.7043	0.0593	0.0003
HPL/SG	61							0.6651	0.0726	0.0115
High HPL/SG ROC	61	73.3	71.0	1.1	99.8	6.7	0.001	0.7215	0.0583	0.0001
SIR Index	62							0.9677	0.0323	0.0000
GAF	59							0.9994	0.0006	0.0000
Hostility	62							0.8710	0.0399	0.0000
Suicidality	62							0.6129	0.0382	0.0016
Family history	62	32.3	71.0	0.5	99.6	1.2	0.783	0.5161	0.0595	0.3934
Developmental delay/ disorder	62	46.7	87.1	1.6	99.7	5.9	0.006	0.6688	0.0555	0.0012
Learning disorder history	62	48.4	87.1	1.7	99.7	6.3	0.004	0.6774	0.0549	0.0006
Head injury	62	48.4	80.6	1.1	99.7	3.9	0.019	0.6452	0.0582	0.0063
Ear infection history	62	32.3	67.7	0.5	99.6	1.0	1.000	0.5000	0.0603	0.5000
Bone conduction abnormality	61	60.0	64.5	0.8	99.7	2.7	0.058	0.6226	0.0631	0.0260
Otoscopy	61	23.3	74.2	0.4	99.5	0.9	0.823	0.4876	0.0560	0.4124

**Heterozygous *MTHFR* 677 CT variant**	*n*	**AUC**	*p*	**OR**	*p*	**Lower**	**Upper**
(Using Logistic regression for Odds Ratio (risk estimate) and						95% CI	95% CI

confidence intervals (CI's)).							
VSOP AgeAdd% ROC (visual speed of processing delay)	60	0.786	0.000	15.278	0.000	4.123	56.608
Low Visual span ROC		0.820	0.000	20.833	0.000	5.622	77.205
Abnormally high score on Distance Vision R ROC	61	0.653	0.005	4.55	0.013	1.376	15.047
Low reverse, digit span score ROC	61	0.773	0.000	16.363	0.000	4.029	66.454
Competing Words % difference ROC (for dichotic listening disorder)	58	0.7893	0.000	19.0	0.000	4.536	79.586
ASOPagediff% ROC (auditory speed of processing delay)	58	0.795	0.000	16.5	0.000	4.354	62.535

S 15. Linear regression analyses for DOI, within three *MTHFR* C677T variants

Biomedical predictors for DOI alone, within the *MTHFR* 677 CC variant, full data set.

All records. MTHFR 677 CC variant biomedicals alone

```
. regress Durationofillnessyrs na da ad_mhma da5hiaa if mthfrpoly==2

      Source |       SS           df       MS      Number of obs   =        58
-------------+----------------------------------   F(4, 53)        =     18.19
       Model |  3709.51684         4  927.379211   Prob > F        =    0.0000
    Residual |   2702.7073        53  50.9944773   R-squared       =    0.5785
-------------+----------------------------------   Adj R-squared   =    0.5467
       Total |  6412.22414        57   112.49516   Root MSE        =     7.141

------------------------------------------------------------------------------
Durationof~s |      Coef.   Std. Err.      t    P>|t|     [95% Conf. Interval]
-------------+----------------------------------------------------------------
          NA |   .3199872   .0646224     4.95   0.000     .1903711    .4496033
          DA |  -.0469889   .0223399    -2.10   0.040    -.0917972   -.0021807
     AD/MHMA|   1.266983   .3951592     3.21   0.002     .4743935    2.059572
   DA X HIAA|     .00338   .0011821     2.86   0.006     .0010089     .005751
       _cons |   1.231476   2.995981     0.41   0.683    -4.777702    7.240653
```

CC variant. `regress Durationofillnessyrs na if mthfrpoly==2 & case==1`

```
      Source |       SS       df       MS              Number of obs =      28
-------------+------------------------------           F(1, 26)      =    6.12
       Model |   568.02316     1   568.02316           Prob > F      =  0.0202
    Residual |  2412.65541    26  92.7944389           R-squared     =  0.1906
-------------+------------------------------           Adj R-squared =  0.1594
       Total |  2980.67857    27  110.395503           Root MSE      =   9.633

------------------------------------------------------------------------------
Durationof~s |      Coef.   Std. Err.      t    P>|t|     [95% Conf. Interval]
-------------+----------------------------------------------------------------
          NA |   .2488562   .1005834     2.47   0.020      .042104    .4556084
       _cons |   6.514023    4.02401     1.62   0.118    -1.757448     14.7855
```

Sensory processing markers for DOI in the whole data set are shown below, where predictive markers were reduced visual span, distance vision impairment and delay in auditory processing speed.

Linear regression for sensory processing predictors for DOI and the *MTHFR* 677 CC variant

```
All records CC variant sensory. regress Durationofillnessyrs vspan dvr6 asoptrim if
mthfrpoly==2
      Source |       SS          df       MS      Number of obs   =        52
-------------+----------------------------------  F(3, 48)        =     15.81
       Model |  1992.60869         3  664.202898  Prob > F         =    0.0000
    Residual |  2016.37207        48  42.0077515  R-squared        =    0.4970
-------------+----------------------------------  Adj R-squared    =    0.4656
       Total |  4008.98077        51  78.6074661  Root MSE         =    6.4813

------------------------------------------------------------------------------
Durationof~s |      Coef.   Std. Err.      t    P>|t|     [95% Conf. Interval]
-------------+----------------------------------------------------------------
Visual span    -3.388428   .7153373    -4.74   0.000     -4.82671   -1.950146
Dist vision R | .299745   .1407094     2.13   0.038     .0168298    .5826602
ASOP % age diff| .094887   .0390318     2.43   0.019     .0164083    .1733656
       _cons |   22.07628   4.361739     5.06   0.000     13.30642    30.84614
```

Linear regression of <u>all</u> predictors for DOI for the *MTHFR* 677 CC variant.

```
Regression for Duration of illness yrs if MTHFR CC
      Source |       SS          df       MS                     Number of obs   =        50
-------------+----------------------------------                 F(7, 42)        =     21.46
       Model |  3085.81157         7  440.830225                 Prob > F        =    0.0000
    Residual |  862.688425        42  20.5402006                 R-squared       =    0.7815
-------------+----------------------------------                 Adj R-squared   =    0.7451
       Total |      3948.5        49  80.5816327                 Root MSE        =    4.5321
------------------------------------------------------------------------------
Durationof~s |          Coef.   Std. Err.      t    P>|t|     [95% Conf. Interval]
-----  --------+----------------------------------------------------------------
          NA |    .131137   .0571088     2.30   0.027     .0158868    .2463872
 Visual span |  -.9495265   .6515324    -1.46   0.152    -2.264372    .3653191
   HPL/creat |   .4680257   .1520334     3.08   0.004     .1612098    .7748416
   Diabetes2 |   15.17467   3.888165     3.90   0.000     7.328038   23.02
 VSOP%ageadd |   .0868472    .036845     2.36   0.023      .012491    .1612034
          AD |   .7715626   .2502485     3.08   0.004     .2665407   1.276585
hypertension|  -5.983589   2.328808    -2.57   0.014    -10.68331   -1.283864
       _cons|   3.221236   4.247426     0.76   0.452    -5.350415   11.79289
```

Linear regression biomedical predictors for DOI for the *MTHFR* 677 TT variant.

For TT variant

Source	SS	df	MS
Model	457.906989	3	152.635663
Residual	5.59301066	2	2.79650533
Total	463.5	5	92.7

Number of obs	=	6
F(3, 2)	=	54.58
Prob > F	=	0.0180
R-squared	=	0.9879
Adj R-squared	=	0.9698
Root MSE	=	1.6723

Durationof~s	Coef.	Std. Err.	t	P>|t|	[95% Conf.	Interval]
Vitamin B 6	.0073858	.0015355	4.81	0.041	.0007792	.0139924
DA X 5HIAA	-.0449125	.0043896	-10.23	0.009	-.0637993	-.0260257
Vit B2 /creat	48.31795	3.885097	12.44	0.006	31.60172	65.03417
_ cons	-3.735599	1.402174	-2.66	0.117	-9.768665	2.297467

Linear regression biomedical predictors for DOI for the *MTHFR* 677 CT variant.

All record CT variant biomedicals alsone

```
. regress Durationofillnessyrs  na vitd vitB12_vitD Histamine if mthfrpoly==1
```

Source	SS	df	MS
Model	3139.81297	4	784.953243
Residual	5078.99211	54	94.0554095
Total	8218.80508	58	141.703536

Number of obs	=	59
F(4, 54)	=	8.35
Prob > F	=	0.0000
R-squared	=	0.3820
Adj R-squared	=	0.3363
Root MSE	=	9.6982

Durationof~s	Coef.	Std. Err.	t	P>|t|	[95% Conf.	Interval]
NA	.2745902	.0643422	4.27	0.000	.1455918	.4035885
vitd	.1852743	.0843721	2.20	0.032	.0161186	.3544301
vitB12_vitD	.5121643	.2106798	2.43	0.018	.0897767	.9345519
Histamine	9.1229	4.290205	2.13	0.038	.5215559	17.72424
_cons	-19.39662	7.069986	-2.74	0.008	-33.57109	-5.222154

Linear regression biomedical predictors for DOI, in relationship to *MTHFR* 677 CT variant.
Linear regression for sensory processing predictors, for DOI in relationship to *MTHFR* 677 CT variant.

All records CT variant sensory

```
. regress Durationofillnessyrs dsbw vspan dvr6  if mthfrpoly==1

      Source |       SS           df       MS      Number of obs   =        60
-------------+----------------------------------   F(3, 56)        =     14.46
       Model |  4006.42458         3  1335.47486   Prob > F        =    0.0000
    Residual |  5171.32125        56  92.3450223   R-squared       =    0.4365
-------------+----------------------------------   Adj R-squared   =    0.4064
       Total |  9177.74583        59  155.555014   Root MSE        =    9.6096

------------------------------------------------------------------------------
Durationof~s |      Coef.   Std. Err.      t    P>|t|     [95% Conf. Interval]
-------------+----------------------------------------------------------------
Digitspan bw |  -2.090973   1.093229    -1.91   0.061    -4.280975    .0990278
  visualspan |  -4.215847   1.086027    -3.88   0.000    -6.391421   -2.040274
distance vis |   .4089338   .2112917     1.94   0.058    -.0143343    .8322019
       _cons |   37.84456   6.184363     6.12   0.000     25.45579    50.23333
------------------------------------------------------------------------------
```

CT variant cases

```
. regress Durationofillnessyrs plasma_HCY na ad_na ad_mhma namhma vitd B2 hplsg
da5hiaa b12_creat Histamine if mthfrpoly==1 & case==1

      Source |       SS           df       MS      Number of obs   =        22
-------------+----------------------------------   F(11, 10)       =      8.11
       Model |  2656.19625        11  241.472386   Prob > F        =    0.0013
    Residual |  297.621931        10  29.7621931   R-squared       =    0.8992
-------------+----------------------------------   Adj R-squared   =    0.7884
       Total |  2953.81818        21  140.658009   Root MSE        =    5.4555

------------------------------------------------------------------------------
Durationof~s |      Coef.   Std. Err.      t    P>|t|     [95% Conf. Interval]
-------------+----------------------------------------------------------------
  plasma_HCY |   3.460276   .9097555     3.80   0.003     1.433214    5.487337
          na |   .5141517   .1442817     3.56   0.005     .1926722    .8356313
       ad_na |  -98.08152   28.55493    -3.43   0.006    -161.7059   -34.45718
     ad_mhma |   4.790443   1.457509     3.29   0.008      1.54291    8.037976
      namhma |  -.0273117   .0057397    -4.76   0.001    -.0401004   -.0145229
        vitd |    .708109   .0945176     7.49   0.000     .4975107    .9187073
          B2 |    1.37133   .2417314     5.67   0.000     .8327187    1.909941
     Hpl/sg |   .0923755   .0281747     3.28   0.008     .0295984    .1551525
  DA X 5hiaa |   -.005015   .0021804    -2.30   0.044    -.0098732   -.0001567
  B12 /creat |   .1031247   .0236326     4.36   0.001     .0504678    .1557815
   Histamine |   30.12513   5.972666     5.04   0.001      16.8172    43.43305
        cons |  -53.72341   12.46929    -4.31   0.002    -81.50671   -25.9401
```

Sensory processing predictors, for DOI in relationship to *MTHFR* 677 CT variant.

Just the cases
CT variant
. regress Durationofillnessyrs vspan dvr6 vsoptrim if mthfrpoly==1 & case==1

Source	SS	df	MS			
Model	1165.82951	3	388.609836	Number of obs	=	28
				F(3, 24)	=	3.61
				Prob > F	=	0.0277
Residual	2580.69728	24	107.529053	R-squared	=	0.3112
				Adj R-squared	=	0.2251
Total	3746.52679	27	138.760251	Root MSE	=	10.37

Durationof~s	Coef.	Std. Err.	t	P>|t|	[95% Conf.	Interval]
vispan	-5.2906	2.203633	-2.40	0.024	-9.838676	-.7425248
distvis	.5313022	.2539714	2.09	0.047	.007131	1.055474
vsop% age add	-.2962108	.1454587	-2.04	0.053	-.5964228	.0040012
_cons	44.11485	12.15763	3.63	0.001	19.02275	69.20696

Biomarker predictors for all variables for DOI in relationship to *MTHFR* 677 CT variant.

For CT variant

Source	SS	df	MS			
				Number of obs	=	47
				F(11, 35)	=	9.25
Model	4059.52186	11	369.047442	Prob > F	=	0.0000
Residual	1395.75474	35	39.8787068	R-squared	=	0.7441
				Adj R-squared	=	0.6637
Total	5455.2766	46	118.592969	Root MSE	=	6.315

Durationofi~s	Coef.	Std. Err.	t	P>|t|	[95% Conf.	Interval]
NA	-16.97548	3.737855	-4.54	0.000	-24.56373	-9.387235
NA/MHMA	-.0070518	.0031462	-2.24	0.031	-.013439	-.0006646
Vitamin B6	-.0591425	.0145763	-4.06	0.000	-.0887339	-.0295511
Vitamin D	.3401709	.0752456	4.52	0.000	.1874142	.4929276
Plasma HCY	-1.675327	.6142962	-2.73	0.010	-2.922415	-.4282392
Histamine + NA	17.24249	3.756067	4.59	0.000	9.617269	24.86771
DA X 5HIAA	.0046706	.0022888	2.04	0.049	.000024	.0093172
vitB12/vitD	.8193047	.2327601	3.52	0.001	.3467767	1.291833
Distance vision R	.6801727	.2109693	3.22	0.003	.2518823	1.108463
Dyslipidaemia	-8.843288	3.000964	-2.95	0.006	-14.93557	-2.751008
BMI	.7295554	.1643833	4.44	0.000	.3958396	1.063271
_cons	-29.67517	8.116896	-3.66	0.001	-46.15335	-13.197

S 16. Spearman's correlates for DOI within *MTHFR* C677T variants.

Significant correlates for DOI within MTHFR CC variant (prevalence 48.5%, Mean DOI 7.18 years), at 95% CI. (low methylation signatures = blue, high methylation signatures = red)

**DOI**	**Variable**	*n*	**Spearman's *rho***	*p*	**Indicator /meaning**
DOI	**Case diagnosis**	60	0.939	0.000	
DOI	**SIR index**	60	0.753	0.000	
DOI	**GAF**	53	-0.793	0.000	
DOI	**CGI**	53	0.850	0.000	
DOI	**SOFAS**	53	-0.790	0.000	
DOI	Family History	59	0.258	0.048	Autosomal recessive inheritance pattern
DOI	Developmental Dis	57	0.676	0.000	
DOI	Learning Disorder	60	0.507	0.000	
DOI	History ear infections	60	0.242	0.076	
DOI	Diabetes (T2)	58	0.316	0.016	
DOI	**Distance vision R ROC**	57	0.370	0.005	**6/6 Sensory Processing deficits**
DOI	**Low visual span ROC**	55	0.698	0.000	
DOI	**VSOP % age add**	54	0.643	0.000	
DOI	**VSOP % age add ROC**	55	0.698	0.000	
DOI	**Low rev digit span ROC**	56	0.528	0.000	
DOI	**ASOP % age diff**	54	0.455	0.001	
DOI	**CW age diff%**	57	-0.527	0.000	
	**CW age diff% ROC**	57	0.541	0.000	
DOI	Suicidality	60	0.594	0.000	**Significant risk over time***

DOI	Hostility	60	0.436	0.000	
DOI	Abuse history	60	0.408	0.001	**37/42 significantly correlating symptoms**
DOI	Ideas of reference/control	60	0.370	0.004	
	**Plus 37/42 significantly correlating symptoms**				
DOI	**DA/HVA**	60	0.121	0.359	**Not sig**
DOI	**DA**	60	0.304	0.018	
DOI	**High DA ROC**	60	0.408	0.001	
DOI	**DA/NA**	60	0.336	0.009	**low Cu or low FAD related ascorbate**
DOI	**NA/MHMA**	58	0.571	0.000	**Low methylation, low FAD, low MAO**
DOI	**High NA/MHMA ROC**	58	0.508	0.000	**Low methylation, low FAD, low MAO**
DOI	**NA**	60	0.661	0.000	**Low methylation, low FAD, low MAO**
DOI	**High NA ROC**	60	0.673	0.000	**Low methylation, low FAD, low MAO**
DOI	**AD/MHMA**	58	0.613	0.000	

DOI	**High AD/MHMA ROC**	58	0.608	0.000	**low FAD, low MAO**
DOI	**NA + AD/MHMA**	58	0.598	0.000	
DOI	**Peak 1/Peak2 amp ROC**	57	0.297	0.035	
DOI	**Peak 1/Peak2 area ROC**	57	0.354	0.007	
	**Peak 1/Peak2 area /creatinine ROC**	57	0.354	0.007	
DOI	**Peak 1-Peak2/creatinine ROC**	57	0.295	0.026	
DOI	**Peak 1 + Peak 2 amplitude**	57	0.259	0.052	
DOI	**Peak 1 + Peak 2 area ROC**	57	0.389	0.000	
DOI	**Vit B12/vit D ROC**	60	0.371	0.004	**low MS, low FAD, low vit D activation**
DOI	**Vitamin D**	60	-0.343	0.007	**low FAD – low activated vit D**
DOI	**Vitamin D ROC**	60	-0.354	0.004	**low FAD – low activated vit D**
DOI	**Low RC folate ROC**	60<br>59	0.285<br>0.252	0.054<br>0.028	**low FAD – low MTHFR - low folate**
DOI	**Vitamin B6**	58	-0.368	0.004	**low FMN, low vitamin B6 activation**
DOI	**Low vit B6 ROC**	60	0.375	0.004	**low FMN, low vitamin B6 activation**
DOI	**Low histamine lab < 0.35**	60	-0.254	0.050	Not associated low histamine (high methylation)

DOI	**HPL/SG ROC**	60	0.292	0.024	
DOI	**HPL/creatinine**	60	0.306	0.017	
DOI	**High HPL/ creatinine ROC**	60	0.262,	0.043	**Low B6, inactive tryptophan pyrrolase (TP) enzyme, residual heme metabolized to HPL.**
DOI	**High 5HIAA ROC**	60	0.475	0.000	**Low B6, low TP, Tryptophan trapping**
DOI	**5 HIAA**	60	0.450	0.000	**Low B6, low TP, Tryptophan trapping**
DOI	**NA/DA**	60	0.596	0.000	**Cu and ascorbate cofactors for DA-> NA**
DOI	**NA/DA ROC**	60	0.573	0.000	**Cu and ascorbate cofactors for DA-> NA**
DOI	**AD/NA**	60	0.336	0.009	
DOI	**High AD/NA ROC**	60	0.402	0.001	**SAMe cofactor for NA -> AD**
DOI	**AD**	60	0.667	0.000	
DOI	**High AD ROC**	60	0.648	0.000	

Correlates for DOI within *MTHFR* TT variant (prevalence 5.2%, mean DOI 5.71 years), at 95% CI. (low methylation signatures = blue, high methylation signatures = red)

Low methylation related to homocysteine trapping (high homocysteine),

Largely dominated by correlates dis-avowing any relationship to 5HIAA as a metabolite of serotonin and tryptophan.

Signature = high AD/NA = surrogate signature of high SAMe.

	**Variable**	*n*	**Spearman's *rho***	*p*	
DOI	MTHFR 677 TT	7	0.956	0.001	
DOI	**Vitamin B2/creatinine ROC**	6	0.908	0.001	
DOI	Peak 2 amplitude / Peak 1 amplitude	6	0.845	0.034	
DOI	Peak 2 area / Peak 1 area	6	0.890	0.034	
DOI	Peak 2 area / Peak 1 area ROC	6	0.980	0.001	
DOI	**Abnormal otoscopy**	7	0.956	0.001	
DOI	HPL/creatinine	7	0.808	0.028	
DOI	DA ROC	7	0.956	0.001	
DOI	DA	7	0.788	0.035	
DOI	NA	7	0.791	0.034	
DOI	HVA/DA	7	1.000	0.000	
DOI	MHMA/NA + AD	7	1.000	0.000	
DOI	ASOP% diff ROC	7	1.000	0.000	
DOI	Low vis span ROC	7	0.698	0.081	
DOI	High DV R ROC	7	0.717	0.070	
DOI	Suicidality	7	0.676	0.096	**marginal sig***
DOI	Depressed mood	7	0.737	0.059	**22/42 significant symptoms**

					**with extended DOI**
DOI	uncooperativeness	7	0.737	0.059	
DOI	**GAF**	7	-0.777	0.040	
DOI	Self-neglect	7	0.872	0.010	
DOI	outside self	7	0.885	0.008	
DOI	bizarre behavior	7	0.885	0.008	
DOI	Unusual thought content	7	0.885	0.008	
DOI	**SOFAS**	7	-0.818	0.025	
DOI	grandiosity	7	0.826	0.023	
DOI	Emotional withdrawal	7	0.836	0.019	
DOI	Social avoidance	7	0.836	0.019	
DOI	Passivity /apathy	7	0.836	0.019	
DOI	Lack pf spontaneous conversation	7	0.836	0.019	
DOI	Anxiety	7	0.923	0.003	
DOI	distractibility	7	0.923	0.003	
DOI	hostility	7	0.923	0.003	
DOI	Blunted affect	7	0.923	0.003	
DOI	Somatic concern	7	0.923	0.003	
DOI	Cognitive disorganization	7	0.956	0.001	
DOI	**Abnormal otoscopy**	7	0.956	0.001	
DOI	**Case-ness**	7	0.956	0.001	
DOI	thought preoccupation	7	0.957	0.001	
DOI	Poor rapport	7	0.957	0.001	
DOI	suspiciousness	7	0.957	0.001	
DOI	delusions	7	0.957	0.001	
DOI	**SIR index**	7	0.957	0.001	

DOI	Hallucinations	7	0.957	0.001	

Significant correlates for DOI within *MTHFR* CT variant (prevalence 46.3%, mean DOI 9.06 years), at 95% CI. (low methylation signatures = blue, high methylation signatures = red)

**DOI**	**Variable**	*n*	**Spearman's *rho***	*p*	**Indicator /meaning**
DOI	**Case diagnosis**	61	0.929	0.000	
DOI	**SIR**	61	0.808	0.000	
DOI	**GAF**	58	-0.844	0.000	
DOI	**CGI**	58	0.907	0.000	
DOI	**SOFAS**	58	-0.824	0.000	
DOI	**Hospital admission frequency**	61	0.806	0.000	
DOI	**Disability support requirement (DSP)**	60	0.835	0.000	
DOI	**Cost care burden**	60	0.862	0.000	
DOI	**Developmental delay/disorder**	60	0.403	0.001	
DOI	**Learning Disorder**	61	0.347	0.006	
DOI	**Subclinical head injury**	61	0.341	0.007	
DOI	**Family History**	59	0.258	0.008	Autosomal recessive inheritance pattern
DOI	**dyslipidemia**	61	-0.226	0.080	
DOI	Distance vision R ROC	60	0.403	0.001	
DOI	Visual span	60	-0.670	0.000	**6/6 sensory processing deficits**
DOI	Low visual span ROC	60	0.684	0.000	
DOI	High VSOP % age add ROC	59	0.478	0.000	
DOI	Reverse digit span	60	-0.567	0.000	

DOI	Low Reverse digit span ROC	60	0.507	0.000	
DOI	ASOP % age diff	57	0.533	0.000	
DOI	ASOP % age diff ROC	57	0.521	0.000	
DOI	CW % age diff	57	-0.502	0.000	
DOI	Low CW % age diff ROC	57	0.590	0.000	
DOI	**hostility**	61	0.575	0.000	**Significantly correlating symptoms 38/42**
DOI	suicidality	61	0.215	0.097	Marginal significance
	**Plus 38/42 significantly correlating symptoms**	61			
DOI	**Abuse history**	61	0.287	0.025	
DOI	**NA/MHMA**	59	0.530	0.000	**Low FAD, low MAO**
DOI	**NA/MHMA ROC**	59	0.553	0.000	**Low FAD, low MAO**
DOI	**NA**	60	0.570	0.000	
DOI	**High NA ROC**	60	0.600	0.000	
DOI	**AD/MHMA**	59	0.428	0.001	**Low FAD, low MAO**
DOI	**AD/MHMA ROC**	59	0.355	0.006	**Low FAD, low MAO**
DOI	**DA**	60	0.338	0.008	
	**High DA ROC**	60	0.339	0.008	
DOI	**DA/NA ROC**	60	0.309	0.016	**Low FAD, low ascorbate**
DOI	**RC folate**	61	-0.258	0.045	
DOI	**Low RC folate ROC**	61	0.280	0.029	**Low FAD, low activated**

					**folate, low methylation**
DOI	Peak 1/Peak 2 amplitude ROC	53	0.390	0.004	
DOI	Peak 1/ peak 2 area ROC	53	0.323	0.018	
	Peak 1/ peak 2 area/creatinine ROC	53	0.361	0.008	
DOI	Area under peak 2 ROC	53	0.403	0.003	
DOI	Serum vit B12	61	0.268	0.018	
DOI	Vitamin B12/RC folate	61	0.321	0.012	
DOI	[Vitamin B12 X % free Cu X homocysteine]/ [zinc X folate X vit B6]	57	0.273	0.040	
DOI	High Serum vitamin B12 ROC	61	0.298	0.020	
DOI	**Low vit B6 ROC**	58	0.230	0.083	**Low FMN, low activated B6**
	**HPL/SG ROC**	57	0.440	0.000	
DOI	**HPL/creatinine**	60	0.631	0.005	**Low B6 with Serine trapping**
DOI	**High HPL/creat ROC**	60	.0.395	0.002	
DOI	**5HIAA**	60	0.241	0.063	**Low B6 with Tryptophan trapping**
DOI	**% Free Cu/Zn ROC**	61	0.260	0.043	**High methylation, low zinc**
DOI	**NA/DA**	60	0.490	0.000	**High methylation, low zinc high Cu**

DOI	NA/DA ROC	60	0.473	0.000	High methylation
DOI	NA/DA	60	0.470	0.000	High methylation, low zinc, high Cu, ascorbate
DOI	NA/DA ROC	60	0.309	0.016	High methylation, low zinc, high Cu , ascorbate
DOI	% free Cu/Zn	60	0.215	0.096	High methylation, low zinc high Cu
DOI	% Free Cu/Zn ROC	60	0.247	0.059	High methylation, low zinc high Cu
DOI	AD	60	0.579	0.000	
DOI	High AD ROC	60	0.601	0.000	
DOI	AD/NA	-	Nil sig	-	
	AD/MHMA				
DOI	High AD/NA ROC	60	0.347	0.007	

S 17. Ranked significant DOI correlates across the three separate MTHFR C677T variants.

Ranked DOI correlates for the *MTHFR* 677 CC variant, significant for long term development of chronicity, severity, and disability.

**VARIABLE**	HEAD INJURY HISTORY	HISTA-MINE ROC	EAR INFEC-TION HIS-TORY	**Low RC Folate ROC**	FAMILY HISTORY	**HPL/Creati-nine ROC**
rho	0.219	0.230	0.242	**0.252**	0.258	**0.262**
p	0.098	0.076	0.076	**0.054**	0.048	**0.043**
n	58	60	55	**59**	59	**60**

mannerism & posturing	DA	HPL/CREAT	Vitamin D	**Peak 1 area/Peak 2 area ROC**	**(High) DV-R6 ROC,**
0.266	0.304	0.306	-0.343	**0.354**	**0.361**
0.040	0.018	0.017	0.007	**0.007**	**0.006**
60	60	60	60	**57**	**57**

**Low Vita-min D ROC**	**B6**	ideas of reference and control	**Vitamin B12/vita-min D ROC**	**Low B6 ROC,**	Low DS B/W ROC,	Disorienta-tion	elated mood
**0.368**	**-0.368**	0.370	**0.371**	**0.375**	0.386	0.386	0.389
**0.006**	**0.004**	0.004	**0.004**	**0.004**	0.003	0.002	0.002
**60**	**58**	60	**60**	**58**	57	60	60

motor hyperactivity	abuse history	somatic concern	grandiosity	**Hostility**	HIAA	guilt	**High HIAA ROC**	**ASOP Age diff% ROC**
0.389	0.408	0.423	0.424	**0.436**	0.450	0.450	**0.475**	**0.455**
0.002	0.001	0.001	0.001	**0.000**	0.000	0.000	**0.000**	**0.001**
60	60	60	60	**60**	60	60	**60**	**54**

LEARNING DISORDER HISTORY	**High ROC (NA/ MHMA)**	**CW% age diff ROC**	Motor retardation	Tension	NA/MHMA	self-neglect	NA/MHMA
0.507	**0.508**	**0.541**	0.552	0.552	0.554	0.571	0.571
0.000	**0.000**	**0.000**	0.000	0.000	0.000	0.000	0.000
60	**60**	**57**	60	60	58	60	58

Depressed mood	**AD + NA /MHMA ROC**	Suicidality	poor impulse control	Unco - operative	AD + NA /MHMA	**AD/MHMA ROC**
0.583	**0.583**	0.594	0.593	0.597	0.598	**0.608**
0.000	**0.000**	0.000	0.000	0.000	0.000	**0.000**
60	**58**	60	60	60	58	**58**

AD/MHMA	abstract thinking impairment	NA	bizarre behavior	**VSOP Age-add% ROC,**	**High AD ROC,**	Anxiety
0.613	0.648	0.661	0.625	**0.643**	**0.648**	0.651
0.000	0.000	0.000	0.000	**0.000**	**0.000**	0.000
58	60	60	60	**54**		60

poor attention	Emotional withdrawal	SIR Index	Unusual Thought content	**SOFAS**	**GAF**
0.730	0.736	0.753	0.787	**- 0.790**	**- 0.793**
0.000	0.000	0.000	0.000	**0.000**	**0.000**
60	60	60	60	**53**	**53**

Disturb volition	**High NA ROC**	DEVELOPMENTAL DELAY DISORDER	Lack spontaneous conversation	Hallucinations	Blunted affect	Passivity / apathy
0.654	**0.673**	0.676	0.677	0.677	0.679	0.682
0.000	**0.000**	0.000	0.000	0.000	0.000	0.000
60	**60**	57	60	60	60	60

distractibility	Social avoidance	Thought. Preoccupation	**Low digit Span ROC**	Cognitive disorganization	poor rapport
0.689	0.692	0.696	**0.698**	0.717	0.718
0.000	0.000	0.000	**0.000**	0.000	0.000
60	60	60	**55**	60	60

Suspiciousness	Judgement and Insight impairment	Delusions	**CGI**	**Case diagnosis**	**Duration of illness years**
0.807	0.822	0.846	**0.850**	**0.939**	**1.000**
0.000	0.000	0.000	**0.000**	**0.000**	
60	60	60	**58**	**60**	

Ranked DOI correlates for homozygous MTHFR 677 TT variant, considered significant in its trajectory from wellbeing to psychosis, with chronicity, severity and disability.

**VARIABLE**	Suicidality	Depressed mood	Uncooperativeness	DA	NA	HPL/ CREAT	**SOFAS**
rho	0.676	0.737	0.764	0.788	0.791	0.808	**-0.818**
p	0.096	0.059	0.046	0.035	**0.034**	**0.028**	**0.025**
n	7	7	7	7	**7**	**7**	**7**

Grandiosity	Social avoidance	Passivity/ apathy	Lack of spontaneous conversation	Emotional withdrawal
0.826	0.836	0.836	0.836	0.836
0.022	0.019	0.019	0.019	0.019
7	7	7	7	7

Self-neglect	0utside of self	bizarre behavior	Unusual Thought content
0.872	0.885	0.885	0.885
0.010	0.008	0.008	0.008
7	7	77	7

Vitamin B2/creatinine ROC	Anxiety	Distract-ability	Hostility	Blunted Affect	Somatic concern
0.908	0.923	0.923	0.923	0.923	0.923
0.001	0.003	0.003	0.003	0.003	0.003
7	7	7	7	7	7

DA ROC	Cognitive Disorganization	**OTOSCOPY abnormality**	**Case-ness**	Thought Preoccupation	Poor Rapport
0.956	0.956	**0.956**	**0.956**	0.957	0.957
0.001	0.001	**0.001**	**0.001**	0.001	0.001
7	7	**7**	**7**	7	7

Suspiciousness	Delusions	**Case identification (diagnosis)**	**SIR Index**	Peak 2 area/Peak 1 area	Hallucinations
0.957	0.957	**0.956**	**0.957**	0.980	0.989
0.001	0.001	**0.001**	**0.001**	0.001	0.000
7	7	**7**	**7**	77	7

**ASOP age %Diff ROC**	**HVA/DA**	**MHMA/NA+AD**	**Duration of illness in years (DOI)**
**1.000**	**1.000**	**1.000**	**1.000**
0.000	**0.000**	**0.000**	
7	**7**	**7**	

Ranked DOI correlates for the heterozygous MTHFR 677 CT variant, significant for long development of chronicity, severity, and disability.

MTHFR +/- and DOI (long)	Suicidality	RC folate	FAMILY HISORY MENTAL ILLNESS	**High % Free Cu:Zn ROC**	HPL/SG	SeB12
rho	0.215	-0.258	**0.258**	**0.260**	0.260	0.268
p	0.097	0.045	**0.008**	**0.043**	0.045	0.037
n	61	61	**59**	**60**	60	61

**High Se B12 ROC**	**(High) Amp projected ROC**	**Low RC Folate ROC**	abuse his-tory	unreal feelings	**High DA ROC**	HEAD INJURY HISTORY
**0.279**	**0.280**	**0.280**	0.287	0.310	**0.339**	0.341
**0.030**	**0.033**	**0.029**	0.025	0.015	**0.008**	0.006
**61**	**58**	**61**	61	61	**60**	61

outside self	LEARNING DISORDER	**AD/ MHMA ROC**	Tension	HPL/ CREAT	blank peri-ods	High DA ROC
0.345	0.347	**0.355**	0.357	0.361	0.372	0393
0.006	0.006	**0.006**	0.005	0.005	0.003	0.002
61	61	**59**	61	60	61	60

HPL/creati-nine ROC	**(High) DV-R6 ROC**	Area under Peak 2 ROC	DEVELOP-MENTAL DELAY / DIS-ORDER	AD/ MHMA	Stereo-typic think-ing
0.395	**0.403**	0.403	0.403	0.428	0.439
0.002	**0.001**	0.004	0.001	0.001	0.000
60	**60**	53	60	59	61

HPL/ Creatine	Motor retardation	Somatic concern	Low DS B/W ROC	**ASOP age diff% ROC**	poor rapport	NA/ MHMA
0.415	0.454	0.458	0.507	**0.517**	0.520	0.530
0.001	0.000	0.000	0.000	**0.000**	0.000	0.000
60	61	61	60	**57**	61	59

**AD+ NA/ MHMA**	**Social avoidance**	**lack of spontaneous conversation**	**Uncooperative**	**Bizarre behavior**	**VSOP Age Add% ROC**
0.552	0.531	0.568	0.534	0.541	**0.542**
0.000	0.000	0.000	0.000	0.000	**0.000**
59	61	61	61	61	**59**

ideas ref/control	elated mood	motor hyperactivity	**High ROC (NA/MHMA**	NA	**Competing words % age diff**
0.543	0.546	0.555	**0.560**	0.570	**-0.502**
0.000	0.000	0.000	**0.000**	0.000	**0.000**
61	61	61	**60**	60	**57**

AD	Disorientation	**High AD ROC**	**High NA ROC**	Passivity /apathy
0.579	0.581	**0.601**	**0.605**	0.605
0.000	0.000	**0.000**	**0.000**	0.000

60	61	**60**	**60**	61

Excitement	Anxiety	Depressed mood	self-neglect	Hostility	Emotional withdrawal	grandiosity	AD+NA/ MHMA ROC
0.617	0.626	0.586	0.643	0.645	0.651	0.657	0.663
0.000	0.000	0.000	0.000	0.000	0.000	0.000	0.000
61	61	61	61	61	61	61	59

Low Visual span ROC	Preoccupation	poor Impulse control	Hallucinations	Blunted Affect	poor attention	abstract thinking Impairment
0.684	0.686	0.691	0.738	0.765	0.766	0.799
0.000	0.000	0.000	0.000	0.000	0.000	0.000
60	61	61	61	61	61	61

Distractability	High Risk of hospital admission	**SIR Index**	Unusual Thought content	**SOFAS**	**GAF**	Delusions
0.694	0.806	**0.808**	0.820	**-0.824**	**-0.844**	0.845
0.000	0.000	**0.000**	0.000	**0.000**	**0.000**	0.000
61	61	**61**	61	**58**	**58**	61

Cognitive Dis-organization	Suspicious-ness	Judgement and Insight Impairment	**CGI**	**Case-ness**	**Duration of ill-ness years**
0.869	0.879	0.902	**0.907**	**0.929**	**1.000**
0.000	0.000	0000	**0.000**	**0.000**	
61	61	61	**58**	**61**	
